# Genetic consequences of cladogenetic vs. anagenetic speciation in endemic plants of oceanic islands

**DOI:** 10.1093/aobpla/plv102

**Published:** 2015-08-26

**Authors:** Koji Takayama, Patricio López-Sepúlveda, Josef Greimler, Daniel J. Crawford, Patricio Peñailillo, Marcelo Baeza, Eduardo Ruiz, Gudrun Kohl, Karin Tremetsberger, Alejandro Gatica, Luis Letelier, Patricio Novoa, Johannes Novak, Tod F. Stuessy

**Affiliations:** 1 Museum of Natural and Environmental History, Shizuoka, Oya 5762, Suruga-ku, Shizuoka-shi, Shizuoka 422-8017, Japan; 2 Departamento de Botánica, Universidad de Concepción, Casilla 160-C, Concepción, Chile; 3 Department of Botany and Biodiversity Research, University of Vienna, Rennweg 14, A-1030 Vienna, Austria; 4 Department of Ecology and Evolutionary Biology and the Biodiversity Institute, University of Kansas, Lawrence, KS 60045, USA; 5 Instituto de Ciencias Biológicas, Universidad de Talca, 2 Norte 685, Talca, Chile; 6 Institute of Botany, Department of Integrative Biology and Biodiversity Research, University of Natural Resources and Life Sciences, Gregor Mendel Straße 33, A-1180 Vienna, Austria; 7 Bioma Consultores S.A., Mariano Sanchez Fontecilla No. 396, Las Condes, Santiago, Chile; 8 Universidad Bernardo O'Higgins, Centro de Investigaciones en Recursos Naturales y Sustentabilidad, General Gana 1702, Santiago, Chile; 9 Jardín Botánico de Viña del Mar, Corporación Nacional Forestal, Camino El Olivar 305, Viña del Mar, Chile; 10 Institute for Applied Botany and Pharmacognosy, University of Veterinary Medicine, Veterinärplatz 1, A-1210 Vienna, Austria; 11 Herbarium, Department of Evolution, Ecology, and Organismal Biology, The Ohio State University, 1315 Kinnear Road, Columbus, OH 43212, USA

**Keywords:** Adaptive radiation, anagenesis, cladogenesis, genetic diversity, phyletic speciation, Robinson Crusoe Islands

## Abstract

Adaptive radiation is a common mode of speciation among plants endemic to oceanic islands. This pattern is one of cladogenesis, or splitting of the founder population, into diverse lineages in divergent habitats. In contrast, endemic species have also evolved primarily by simple transformations from progenitors in source regions. This is anagenesis, whereby the founding population changes genetically and morphologically over time primarily through mutation and recombination. Gene flow among populations is maintained in a homogeneous environment with no splitting events. Genetic consequences of these modes of speciation have been examined in the Juan Fernández Archipelago, which contains two principal islands of differing geological ages. This article summarizes population genetic results (nearly 4000 analyses) from examination of 15 endemic species, involving 1716 and 1870 individuals in 162 and 163 populations (with amplified fragment length polymorphisms and simple sequence repeats, respectively) in the following genera: *Drimys* (Winteraceae), *Myrceugenia* (Myrtaceae), *Rhaphithamnus* (Verbenaceae), *Robinsonia* (Asteraceae, Senecioneae) and *Erigeron* (Asteraceae, Astereae). The results indicate that species originating anagenetically show high levels of genetic variation within the island population and no geographic genetic partitioning. This contrasts with cladogenetic species that show less genetic diversity within and among populations. Species that have been derived anagenetically on the younger island (1–2 Ma) contain less genetic variation than those that have anagenetically speciated on the older island (4 Ma). Genetic distinctness among cladogenetically derived species on the older island is greater than among similarly derived species on the younger island. An important point is that the total genetic variation within each genus analysed is comparable, regardless of whether adaptive divergence occurs.

## Abstract (precis)

This paper presents for the first time a comparison of the genetic consequences of two different types of speciation in plants of an oceanic island. Genetic data, using two different DNA methods, were obtained from more than 4,000 plants from the two major islands of the Juan Fernández Archipelago (Chile). Results show that some immigrant populations undergo major splitting events and harbor limited genetic diversity within each evolving line. In contrast, other immigrant populations establish and enlarge, but they never split, hence accumulating higher levels of genetic diversity.

## Introduction

Oceanic islands have long stimulated biologists to investigate patterns and processes of evolution (e.g. Darwin 1842; Wallace 1881; Whittaker and Fernández-Palacios 2007; Bramwell and Caujapé-Castells 2011). These isolated land masses, far from continental source areas, offer opportunities for determining origins of immigrants and their evolutionary history after establishment. The low probability of long-distance dispersal and successful colonization, the reduction of genetic variation in founding populations and the challenges of adaptation to new environments are all features that combine to affect processes of evolution in island archipelagos, particularly speciation.

**Figure 1.**
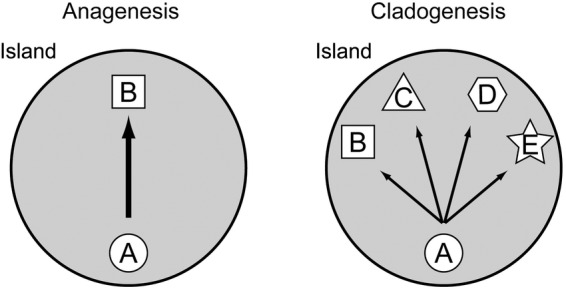
Diagram of the two principal modes of speciation in oceanic islands.

One dimension of speciation in island plants that has received considerable attention is adaptive radiation (Carlquist 1974; Whittaker and Fernández-Palacios 2007; Rundell and Price 2009). This is a process that begins with dispersal from the original immigrant population into different habitats on the same or neighbouring island. This isolation leads to divergence of the new segregate populations, each becoming rapidly adapted to divergent habitats (Schluter 2001), such that eventually new species are recognized taxonomically. This general process of speciation is usually diagrammed (Fig. 1) as splitting events or cladogenesis (Rensch 1959). A number of dramatic species complexes have developed in oceanic islands through adaptive radiation, such as illustrated by the lobelioids (Givnish *et al.* 2009) and silverswords (Carlquist *et al.* 2003) in Hawaii, *Aeonium* (Liu 1989; Jorgensen and Olesen 2001) and *Echium* (Böhle *et al*. 1996) in the Canary Islands and *Scalesia* (Eliasson 1974) in the Gálapagos archipelago.

In addition to speciation via adaptive radiation (involving cladogenesis), another process, anagenesis (Fig. 1), has recently been emphasized (Stuessy *et al*. 1990, 2006; Whittaker *et al.* 2008). Some immigrant populations, especially when arriving on an island with limited ecological opportunity, proliferate in size and accumulate genetic diversity mainly through mutation and recombination. After many generations (perhaps over a million or more years), genetic changes result in different morphology that may be treated as a distinct species. This process has been labelled anagenetic speciation (Stuessy *et al*. 2006), being one type of progenitor-derivative speciation (Crawford 2010). It has been estimated that at least one-quarter of all endemic plant species of oceanic islands have originated via anagenesis (Stuessy *et al*. 2006).

Some studies have been published on the genetic consequences of cladogenesis in endemic plants of different archipelagos. Böhle *et al*. (1996) examined chloroplast sequence variation among endemic species of *Echium* (Boraginaceae) of the Canary Islands, showing very little nucleotide divergence even though the morphological variation is striking. Likewise, Baldwin (2003) examined internal transcribed spacer regions of nuclear ribosomal DNA (ITS) variation among species of the Hawaiian silverswords (Asteraceae) and again, limited sequence variation was seen. The general result from these, and other studies, is that during cladogenesis, the immigrant population becomes fragmented, with each segment containing a limited range of genetic variation in comparison with the continental progenitor population (Baldwin *et al*. 1998). Maximum morphological divergence occurs but with low levels of observable genetic diversity (Frankham 1997). There is some evidence (Perugganan *et al*. 2003) that the genetic changes responsible for the morphological adaptations involve alterations in regulatory rather than structural genes.

Results so far with anagenesis show a strikingly different pattern. Most of the investigations have been done on endemic species of Ullung Island, in which at least 88 % of the endemic species have originated anagenetically (Stuessy *et al*. 2006). The island is young (1.8 Ma; Kim 1985), of low elevation (<1000 m) and relatively ecologically uniform (Yim *et al*. 1981). Pfosser *et al*. (2005), using amplified fragment length polymorphisms (AFLPs), examined island and Japanese populations of *Dystaenia takesimana* and *D. ibukiensis*, respectively, and the results showed high levels of genetic variation within *D. takesimana* in comparison with *D. ibukiensis*. Similar results have been obtained in assessing the origin of *Acer takesimensis* and *A. okomotoanum* (Takayama *et al*. 2012, 2013*a*). Because there is no partitioning of the immigrant population, it survives and proliferates, during which time it accumulates genetic variation through mutation and recombination. Eventually, the level of genetic diversity may even equal (or surpass) that observed in parental source populations (Stuessy 2007).

Because the above studies have been done on different genera in different island archipelagos, it would be useful to compare the genetic consequences of both types of speciation within groups of the same island system, preferably within the same island. In this fashion, more direct comparisons can be made because the general environment is the same. Important, obviously, is to locate plant groups that have originated via both anagenesis and cladogenesis within the same archipelago. A good choice for examining the genetic consequences of anagenesis and cladogenesis in endemic plants of oceanic islands is the Juan Fernández Archipelago, Chile. Approximately 64 % of the species have originated by cladogenesis and 36 % by anagenesis (Stuessy *et al*. 2006). From another perspective, it is estimated that 70 % of the *colonists* to the islands have diverged anagenetically, in contrast to only 30 % that have diverged via adaptive radiation (Stuessy *et al*. 1990).

**Figure 2.**
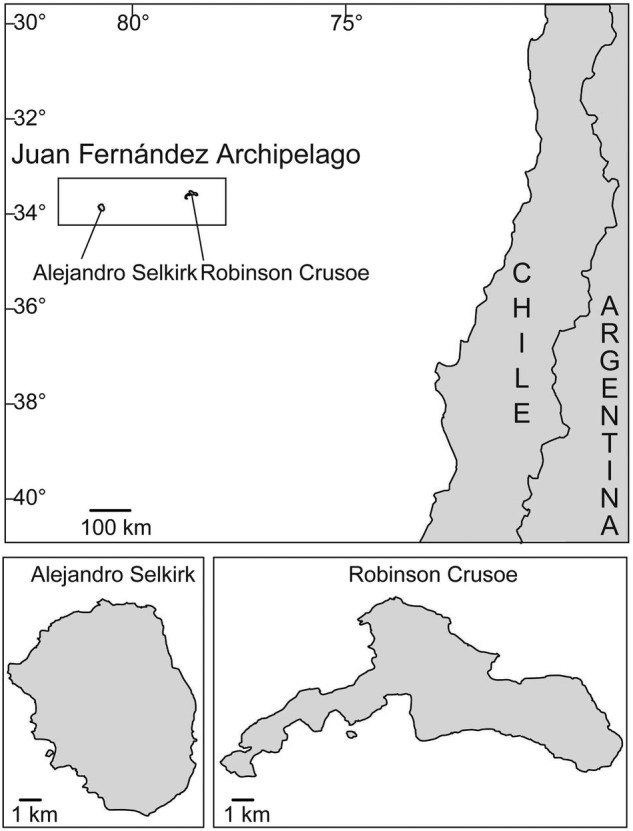
Location of the Juan Fernández Archipelago and its two major islands, Alejandro Selkirk (= Masafuera) and Robinson Crusoe (= Masatierra).

The Juan Fernández Archipelago consists of two major islands (Fig. 2): Robinson Crusoe (= Masatierra), located 667 km west of continental Chile at 33°S latitude, and Alejandro Selkirk (= Masafuera) situated 181 km further westward into the Pacific Ocean. The former is known to be ∼4 million years old and the latter 1–2 million years old (Stuessy *et al*. 1984). At present, these two islands are approximately the same size of 50 km^2^ (Stuessy 1995). The flora is small, containing 78 native and 135 endemic vascular plant species (Danton *et al*. 2006). From a biogeographic standpoint, this setting is particularly favourable for generating initial hypotheses, because the near island (Robinson Crusoe) is also the older one, making it highly probable as the initial site for colonization of most groups. Furthermore, the older island is hypothesized to have been much larger when formed (Stuessy *et al*. 1998), making it a bigger target for dispersal from the mainland.

Numerous molecular markers now exist for assessing genetic variation within and among populations (Lowe *et al.* 2004). Amplified fragment length polymorphisms (Vos *et al*. 1995) have been used effectively to provide an overall evaluation of population genetic diversity (Tremetsberger *et al*. 2003; López-Sepúlveda *et al*. 2013*a*). These are treated as dominant markers and hence cannot be employed to determine allelic frequencies. An appropriate co-dominant and polymorphic marker that does allow allelic calculations are nuclear microsatellites or simple sequence repeats (SSRs). The challenge with this marker is to develop primers for locating sequences within the genome for comparison. Next-generation sequencing (NGS) methods are now available that allow this to be done much more easily and at reasonable cost (Takayama *et al*. 2011, 2013*b*). Numerous successful applications of SSRs have shown their efficacy to reveal genetic variation at the population level (Gleiser *et al*. 2008; Kikuchi *et al*. 2009; López-Sepúlveda *et al*. 2013*b*).

Studies using AFLPs and SSRs have already been published on a number of endemic taxa of the Juan Fernández Archipelago, representing groups that have undergone speciation via cladogenesis and anagenesis. The largest (and endemic) genus that has been investigated is *Robinsonia* (Asteraceae; Takayama *et al*. 2015), which has seven species on Robinson Crusoe Island that have originated cladogenetically and one on Alejandro Selkirk Island that has evolved anagenetically. The genus *Erigeron* (Asteraceae; López-Sepúlveda *et al*. 2015) has six species that evolved cladogenetically on the younger island, Alejandro Selkirk. These two genera were selected because *Robinsonia* has speciated primarily via cladogenesis on the older island, and *Erigeron* has done so on the younger island. Regarding anagenesis, studies have been completed on *Drimys confertifolia* (Winteraceae; López-Sepúlveda *et al*. 2014) and *Rhaphithamnus venustus* (Verbenaceae; P. López-Sepúlveda, K. Takayama, D. J. Crawford, J. Greimler, P. Peñailillo, M. Baeza, E. Ruiz, G. Kohl, K. Tremetsberger, A. Gatica, L. Letelier, P. Novoa, J. Novak, T. F. Stuessy, submitted for publication), which occur on both islands of the archipelago. Investigations have also been completed on *Myrceugenia* (Myrtaceae; López-Sepúlveda *et al*. 2013*b*), which contains one endemic species on each of the islands. The available genetic data to date, therefore, come from 15 endemic species, plus 4 close continental relatives, summing to 1870 individuals in 163 populations.

The purposes of this article are to (i) summarize published data from AFLP and SSR investigations on endemic species of the genera *Drimys*, *Myrceugenia*, *Rhaphithamnus*, *Robinsonia* and *Erigeron*; (ii) compare and contrast differences in genetic diversity in groups that have undergone anagenetic or cladogenetic speciation and (iii) discuss the importance of considering modes of speciation for understanding levels of genetic diversity within endemic species of oceanic archipelagos.

## Methods

The data summarized here (Table 1) provide the first comprehensive genetic comparisons (with AFLPs and SSRs) in the Juan Fernández Archipelago of species that have evolved by anagenesis and cladogenesis, based on consistent samplings, laboratory methods and modes of analysis. A number of earlier studies utilizing isozymes and DNA sequences have examined genetic variation in endemic species of these islands (e.g. Crawford *et al.* 1998, 2001*a*), but these investigations were not focussed on comparing modes of speciation. Genera in the present studies were selected for their representation of anagenesis and cladogenesis and for their occurrence on the two islands of different geological ages. The samples were collected during expeditions in February 2010 and 2011 from 1870 individuals in 163 populations in 15 endemic species, hence representing 14 % of the endemic angiosperms in the archipelago. The samples provide very good geographic coverage of populations over the landscape in both islands. The term population, as used here in the sense of sampling, refers to groups of individuals that were clearly delimited spatially in the field. The number of individuals analysed per population ranged from 1 to 31. The voucher data for these samples and details of data gathering and analysis are given in the respective publications.

**Table 1.** Summary of measures of genetic diversity in endemic species of the Juan Fernández Archipelago that have originated by anagenesis or cladogenesis. All average values. Data from López-Sepúlveda *et al.* (2013*a*, *b*, 2014), Takayama *et al.* (2015) and P. López-Sepúlveda, K. Takayama, D. J. Crawford, J. Greimler, P. Peñailillo, M. Baeza, E. Ruiz, G. Kohl, K. Tremetsberger, A. Gatica, L. Letelier, P. Novoa, J. Novak, T. F. Stuessy, submitted for publication. TNB, total number of bands (fragments); PPB, percentage of polymorphic bands; SDI, Shannon Diversity Index; AGDOL, average gene diversity over loci; RI, rarity index; *H*_O_, observed proportion of heterozygotes; *H*_E_, expected proportion of heterozygotes; *N*_A_, number of alleles per locus; *F*_IS_, inbreeding coefficient; *A*_R5_, allelic richness standardized by five individuals; RC, Robinson Crusoe Island; AS, Alejandro Selkirk Island.

Species	AFLPs							Microsatellites (SSRs)						
	No. of pops.	No. of plants	TNB	PPB	SDI	AGDOL	RI	No. of pops.	No. of plants	*H*_O_	*H*_E_	*N*_A_	*F*_IS_	*A*_R5_
Anagenesis														
*D. confertifolia* (RC)	16	183	557	96.5	125.3	0.26	1.96	16	181	0.48	0.68	9.00	0.29	4.12
*D. confertifolia* (AS)	15	96	538	96.5	114.3	0.23	2.26	15	80	0.35	0.51	6.38	0.26	3.24
*D. confertifolia* (combined RC and AS)	31	279	576	100	134.7	0.28	2.06	31	261	0.44	0.68	9.88	0.33	4.13
*M. fernandeziana* (RC)	18	211	371	100	74.6	0.23	1.76	18	231	0.38	0.49	10.08	0.19	3.38
*M. schulzei* (AS)	13	129	417	100	96.2	0.28	3.39	13	155	0.39	0.61	10.33	0.35	3.79
*R. venustus* (RC)	20	143	440	99.3	96.4	0.25	2.80	20	140	0.17	0.23	4.22	0.31	1.83
*R. venustus* (AS)	4	18	271	57.3	60.8	0.18	2.34	4	11	0.30	0.34	2.33	0.13	2.12
*R. venustus* (combined RC and AS)	24	161	443	100	98.7	0.26	2.75	24	151	0.18	0.28	4.56	0.40	2.04
*R. masafuerae* (AS)	5	9	344	41.4	84.1	0.15	2.90	5	7	0.36	0.43	3.50	0.17	3.08
Cladogenesis														
*Robinsonia gayana* (RC)	10	123	592	77.2	111.0	0.16	2.39	10	134	0.34	0.42	6.30	0.28	3.04
*R. gracilis* (RC)	5	75	515	63.2	97.3	0.15	2.68	5	87	0.28	0.39	3.50	0.24	2.26
*R. evenia* (RC)	6	73	586	73.4	112.0	0.17	3.18	6	86	0.21	0.26	2.80	0.21	1.87
*R. saxatilis* (RC)	1	5	267	29.0	67.0	0.14	1.99	1	5	0.30	0.26	2.10	−0.22	2.10
*Robinsonia* (combined all RC species)	22	276	765	100	183.7	0.26	2.77	22	312	0.28	0.66	8.40	0.61	3.97
*Robinsonia* (combined all species)	27	285	766	100	265.0	0.26	2.68	27	319	0.29	0.67	8.70	0.61	4.02
*E. fernandezianus* (RC)	13	240	403	90.3	70.7	0.20	0.58	13	271	0.21	0.29	4.20	0.31	2.17
*E. fernandezianus* (AS)	19	172	426	95.3	81.1	0.23	0.81	19	200	0.17	0.50	7.50	0.72	3.27
*E. fernandezianus* (combined RC and AS)	32	412	433	97.5	81.7	0.23	0.68	32	471	0.20	0.40	8.00	0.64	2.86
*E. ingae* (AS)	2	21	315	61.3	62.0	0.18	0.62	2	25	0.20	0.34	2.90	0.55	2.04
*E. luteoviridis* (AS)	2	25	334	61.5	60.2	0.18	0.99	2	25	0.05	0.31	3.10	0.72	2.19
*E. rupicola* (AS)	9	175	377	81.8	69.5	0.20	0.67	9	211	0.17	0.36	4.40	0.57	2.43
*E. turricola* (AS)	3	10	269	49.3	57.6	0.19	0.50	3	10	0.24	0.53	3.40	0.57	2.94
*E. stuessyi* (AS)	1	8	306	66.7	82.4	0.28	0.81	2	11	0.20	0.25	2.10	0.53	1.89
*Erigeron* (combined all AS species)	36	411	443	100	95.1	0.26	0.74	37	482	0.17	0.62	9.20	0.76	2.85
*Erigeron* (combined all species)	49	651	444	100	94.2	0.26	0.68	50	753	0.18	0.56	9.50	0.73	3.46
Total and averages														
Anagenesis	91	789	419.7	84.4	93.1	0.23	2.49	91	805	0.35	0.47	6.55	0.24	3.08
Cladogenesis	71	927	399.1	68.1	79.2	0.19	1.38	72	1065	0.2	0.4	3.8	0.41	2.38
Robinson Crusoe	89	1053	466.4	78.6	94.3	0.19	2.17	89	1135	0.30	0.38	5.28	0.20	2.60
Alejandro Serkirk	73	663	359.7	71.1	76.8	0.21	1.53	74	735	0.24	0.42	4.59	0.46	2.70
Anagenesis (RC)	54	537	456.0	98.6	98.8	0.24	2.17	54	552	0.34	0.47	7.77	0.26	3.11
Anagenesis (AS)	37	252	392.5	73.8	88.9	0.21	2.72	37	253	0.35	0.47	5.64	0.23	3.06
Cladogenesis (RC)	35	516	472.6	66.6	91.6	0.16	2.16	35	583	0.27	0.32	3.78	0.17	2.29
Cladogenesis (AS)	36	411	337.8	69.3	68.8	0.21	0.73	37	482	0.17	0.38	3.90	0.61	2.46

Briefly, the following approaches were used for AFLPs. Four or six selective primer combinations were chosen. Numerous (24–85) primer trials were run with each genus to determine the best combination of primers for good resolution of individuals and populations. Data were obtained on an automated DNA sequencer (ABI 3130xl, Applied Biosystems, Waltham, MA, USA). Scoring was done using GeneMarker ver. 1.85 (SoftGenetics, State College, PA, USA). For analysis of AFLP data, the programs Arlequin 3.5.1.2 (Excoffier *et al*. 2005), FAMD ver. 1.25 (Schlüter and Harris 2006), R-Script AFLPdat (Ehrich 2006) and SPSS ver. 15.0 (SPSS; IBM, Armonk, NY, USA) were employed to determine total number of fragments (TNB), percentage of polymorphic fragments (PPB), Shannon Diversity Index (SDI), average gene diversity over loci (AGDOL) and rarity index (RI).

For SSRs, NGS methods (Takayama *et al*. 2011) were used to generate 6–12 loci, selected for their repeatability and scoring convenience. Polymerase chain reaction-amplified fragments were also run on the same automated sequencer and scored with GeneMarker ver. 1.85. Data analysis involved using GENEPOP 4.0 (Raymond and Rousset 1995), Micro-Checker 2.2.3 (van Oosterhout *et al*. 2004), FSTAT 2.9.3.2 and GENALEX 6 (Peakall and Smouse 2006). These allow analyses for observed proportion of heterozygotes (*H*_O_), expected proportion of heterozygotes (*H*_E_), number of alleles per locus (*N*_A_), inbreeding coefficient (*F*_IS_) and allelic richness standardized by five individuals (*A*_R5_).

The overall pattern of higher genetic diversities in anagenetically derived species in comparison with cladogenetically derived ones was examined by a Student's *t*-test (average TNB, PPB, SDI, AGDOL and RI in AFLPs, and *H*_O_, *H*_E_, *N*_A_ and *A*_R5_ in SSRs) and shown in Table 2. To improve normality of *H*_O_ and *H*_E_, a square-root transformation was applied. The overall patterns of higher genetic diversities in Robinson Crusoe Island (old) than Alejandro Selkirk Island (new) were also examined in the same way. The effects of two factors (speciation mode and island) and their interaction were analysed in a two-way ANOVA in R version 3.0.0 (R Core Team 2013) and shown in Table 3.

**Table 2.** Summary of statistical tests based on Table 1. TNB, total number of bands (fragments); PPB, percentage of polymorphic bands; SDI, Shannon Diversity Index; AGDOL, average gene diversity over loci; RI, rarity index; *H*_O_, observed proportion of heterozygotes; *H*_E_, expected proportion of heterozygotes; *N*_A_, number of alleles per locus; *A*_R5_, allelic richness standardized by five individuals. Bold font indicates significant values (*P* < 0.05).

	High genetic diversity in anagenetically derived species	High genetic diversity in Robinson Crusoe Island species
AFLPs		
TNB	0.351	**0.024**
PPB	0.086	0.235
SDI	0.101	**0.045**
AGDOL	**0.050**	0.227
RI	**0.004**	0.085
SSRs		
*H*_O_	**0.006**	0.132
*H*_E_	0.061	0.236
*N*_A_	**0.040**	0.308
*A*_R5_	**0.038**	0.388

**Table 3.** Summary of two-way ANOVA based on Table 1. TNB, total number of bands (fragments); PPB, percentage of polymorphic bands; SDI, Shannon Diversity Index; AGDOL, average gene diversity over loci; RI, rarity index; *H*_O_, observed proportion of heterozygotes; *H*_E_, expected proportion of heterozygotes; *N*_A_, number of alleles per locus; *A*_R5_, allelic richness standardized by five individuals. For all *F*-values, the degree of freedom was 1. Bold font indicates significant values (*P* < 0.05).

	Factor	*F*-value	*P*-value
AFLPs			
TNB	Island	4.78	**0.046**
	Speciation mode	0.22	0.645
	Island vs. speciation mode	0.51	0.489
PPB	Island	0.67	0.427
	Speciation mode	2.60	0.129
	Island vs. speciation mode	2.05	0.174
SDI	Island	3.61	0.078
	Speciation mode	2.36	0.147
	Island vs. speciation mode	0.47	0.504
AGDOL	Island	0.85	0.372
	Speciation mode	4.09	0.063
	Island vs. speciation mode	4.67	**0.048**
RI	Island	4.63	**0.049**
	Speciation mode	13.71	**0.002**
	Island vs. speciation mode	10.53	**0.006**
SSRs			
*H*_O_	Island	2.03	0.176
	Speciation mode	11.65	**0.004**
	Island vs. speciation mode	1.64	0.221
*H*_E_	Island	0.47	0.502
	Speciation mode	3.44	0.085
	Island vs. speciation mode	0.19	0.671
*N*_A_	Island	0.47	0.502
	Speciation mode	3.44	0.085
	Island vs. speciation mode	0.19	0.671
*A*_R5_	Island	0.10	0.752
	Speciation mode	4.54	0.051
	Island vs. speciation mode	0.11	0.744

Data from both AFLPs and microsatellites were further analysed by assessing genetic distance (Nei *et al*. 1983) with the NeighborNet algorithm (Bryant and Moulton 2004) implemented by SplitsTree4 ver. 4.10 (Huson and Bryant 2006) and Population 1.2.30 (Langella 1999), respectively.

**Figure 3.**
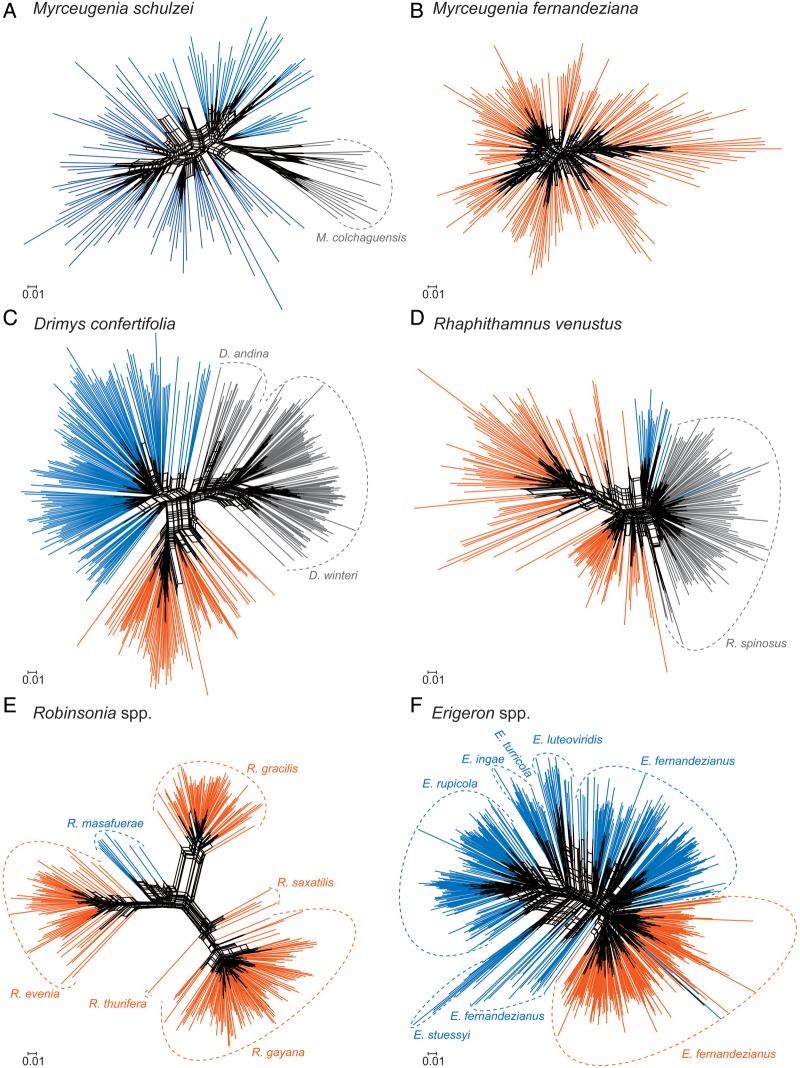
SplitsTree NeighborNet showing genetic relationships based on AFLPs among individuals in endemic species of *Myrceugenia* (A and B), *Drimys* (C), *Rhaphithamnus* (D), *Robinsonia* (E) and *Erigeron* (F) in the Juan Fernández Archipelago. Closely related continental relatives are also shown in A, C and D. Orange = species and populations on Robinson Crusoe Island; blue = on Alejandro Selkirk Island and black = on the or islands continent.

**Figure 4.**
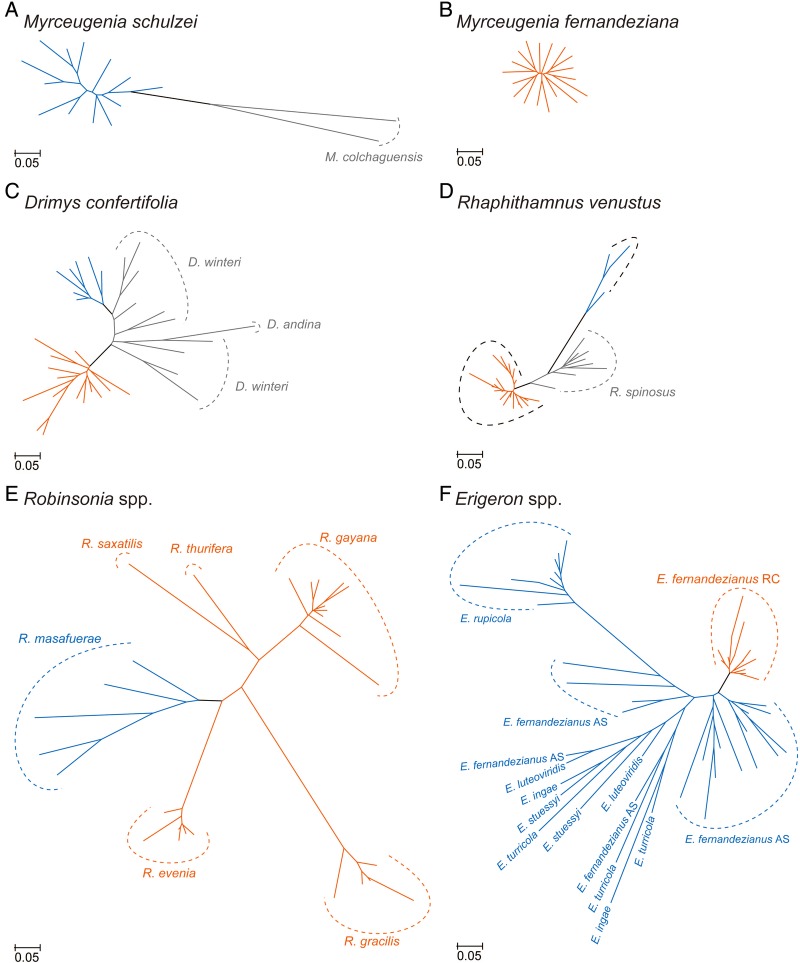
Neighbour-joining tree showing genetic relationships based on SSRs among populations in endemic species of *Myrceugenia* (A and B), *Drimys* (C), *Rhaphithamnus* (D), *Robinsonia* (E) and *Erigeron* (F) in the Juan Fernández Archipelago. Closely related continental relatives are also shown in A, C and D. Orange = species and populations on Robinson Crusoe Island; blue = on Alejandro Selkirk Island and black = on the continent.

**Figure 5.**
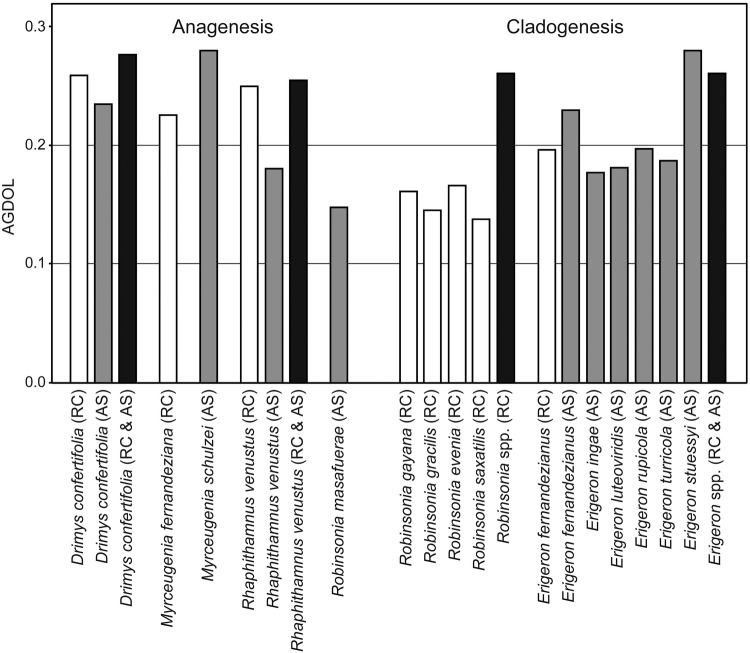
Summary of genetic diversities, AGDOL, within the endemic species of *Drimys*, *Myrceugenia* and *Rhaphithamnus* having originated by anagenesis, and *Robinsonia* and *Erigeron* having been derived through cladogenesis. *Robinsonia masafuerae* from the younger island is also an anagenetic derivative from the cladogenetic complex of *Robinsonia* on the older island. AS, Alejandro Selkirk Island; RC, Robinson Crusoe Island. White bar indicates an endemic species in RC, grey bar an endemic species in AS and black bar multiple species or islands combined.

For this article, to allow ease of visual comparisons of results among the species, emphasis has been placed on selected graphic presentations. SplitsTree NeighborNet was employed with the AFLP data, and the results are given in a series of graphs (Fig. 3). Neighbour-joining based on genetic distance was used for analysis of the SSRs, and simplified networks were used to show relationships among the populations (Fig. 4). For summary comparisons of genetic diversity among species, AGDOL was used with the AFLP data (Fig. 5). Not all calculated values for all original populations are presented or discussed in this review. The reader is referred to the original publications for additional methods and data.

## Results

The results from the AFLP and SSR data analyses are given in Tables 1–4 and shown graphically in Figs 3–5. In general, the results from the two sources of genetic data are similar, with some exceptions, reinforcing confidence in the patterns seen. These data will be presented in context of the two modes of speciation, anagenesis and cladogenesis, but with attention also to the different ages of the islands. Robinson Crusoe Island is ∼4 million years old and Alejandro Selkirk 1–2 million (Stuessy *et al*. 1984).

**Table 4.** Generalized comparison of the levels of genetic diversity obtained with AFLPs and SSRs from species that have originated via anagenesis and cladogenesis on the two islands of the Juan Fernández Archipelago. See Table 1 for the actual data. RC, Robinson Crusoe Island; AS, Alejandro Selkirk Island.

	Anagenesis		Cladogenesis	
	RC	AS	RC	AS
AFLPs				
Total number of bands (TNB)	High	Medium	High	Low
Percentage of polymorphic bands (PPB)	High	Low	Low	Low
Shannon Diversity Index (SDI)	High	Medium high	Medium high	Low
Average gene diversity over loci (AGDOL)	High	Medium high	Low	Medium high
Rarity index (RI)	Medium	High	Medium	Very low
Microsatellites (SSRs)				
Observed proportion of heterozygotes (*H*_O_)	High	High	Medium	Low
Expected proportion of heterozygotes (*H*_E_)	High	High	Medium	Medium
Number of alleles per locus (*N*_A_)	High	Medium	Low	Low
Inbreeding coefficient (*F*_IS_)	Low	Low	Low	High
Allelic richness (*A*_R5_)	High	High	Low	Low

### Anagenesis

The results from analysis of species that have evolved anagenetically include those from *Myrceugenia fernandeziana*, *M. schulzei*, *Robinsonia masafuerae*, *D. confertifolia* and *R. venustus*. The first species occurs only on the older island, the second and third species only on the younger island and the last two on both islands. A number of points seem evident. First, all anagenetically derived species show considerable levels of genetic diversity (Table 1, and Figs 3 and 5), and none of them shows geographic patterns over the island landscape (López-Sepúlveda *et al*. 2013*b*, 2014, P. López-Sepúlveda, K. Takayama, D. J. Crawford, J. Greimler, P. Peñailillo, M. Baeza, E. Ruiz, G. Kohl, K. Tremetsberger, A. Gatica, L. Letelier, P. Novoa, J. Novak, T. F. Stuessy, submitted for publication). This is what might be expected from the predictions regarding anagenesis based on previous studies. Even more interesting, perhaps, is that the amount of genetic diversity differs in species on the two islands of different ages. In *D. confertifolia*, and *R. venustus*, which occur on both islands, one sees in both cases more genetic diversity (SDI) in populations on the older island than on the younger island except for estimates of SSRs in *R. venustus* (Table 1). The explanation of these data may relate to the time available for a genetic change to take place. Because Alejandro Selkirk Island is no more than 1–2 million years old, this must be the maximum time available for population divergence to take place. With anagenetically evolved species, all factors being equal, genetic variation increases through time, and this can be seen in the species investigated.

One case of anagenesis in the archipelago also merits comment. *Robinsonia masafuerae* is a species that appears to have speciated from *R. evenia*, with which it has been closely associated in all studies so far (Crawford *et al*. 1993*a*; Sang *et al*. 1995; Takayama *et al*. 2015). Previous investigations on ITS 1 and 2 in *Robinsonia* (Sang *et al*. 1995) have shown sequence divergence between *R. evenia* and *R. masafuerae* as only 0.0063 (two base substitutions). Although one cannot place an absolute time on this divergence, it is the lowest level among any pair of species in the genus, which correlates well with the youthful geological age of Alejandro Selkirk Island. Genetic variation in *R. masafuerae* is much lower from AFLP data than in *R. evenia* from Robinson Crusoe (Table 1 and Fig. 5), but in SSRs, the pattern reverses with the anagenetically derived species, *R. masafuerae*, showing more variation than any single one of the cladogenetically originated species on Robinson Crusoe (Table 1).

It is also possible to make comparisons between populations of continental progenitors with endemic island derivatives. In the case of *Myrceugenia schulzei*, the closest continental congener is *M. colchaguensis* (Landrum 1981*a*, *b*; Ruiz *et al*. 2004). Although the sampling of populations on the continent is limited to two populations, the amount of genetic diversity is particularly low as shown by AFLP data, although somewhat higher with SSRs (López-Sepúlveda *et al*. 2013*b*). Although *M. schulzei* is known only on the younger island, it did not diverge from *M. fernandeziana* on the older island because the two are unrelated (Murillo-Aldana *et al*. 2012), so much so that the latter has now been transferred to another genus (*Nothomyrcia*; Murillo-Aldana and Ruiz 2011). With *D. confertifolia*, comparisons with *D. winteri* and *D. andina* show less genetic variation in the two latter species as seen from AFLPs and SSRs (López-Sepúlveda *et al*. 2014). In *R. venustus*, which is a congener of *R. spinosus* (the only other known species in the genus; Moldenke 1937; Crawford *et al*. 1993*b*), the amount of genetic diversity is again greater in the population on Robinson Crusoe Island than documented on the continent, although considerably lower in the population on Alejandro Selkirk (P. López-Sepúlveda, K. Takayama, D. J. Crawford, J. Greimler, P. Peñailillo, M. Baeza, E. Ruiz, G. Kohl, K. Tremetsberger, A. Gatica, L. Letelier, P. Novoa, J. Novak, T. F. Stuessy, submitted for publication). These results support the concept that over time, considerable genetic variation can accumulate in anagenetically derived populations, so much so that the degree of variation can approximate and even surpass that in the progenitor source populations.

### Cladogenesis

Two of the largest genera of the archipelago are *Robinsonia* with eight endemic species and *Erigeron* with six. Both are in Asteraceae, although unrelated and placed in different tribes (Senecioneae vs. Astereae, respectively). *Robinsonia* has adaptively radiated on Robinson Crusoe Island during the past 4 million years (maximum value) and *Erigeron* has done so on Alejandro Selkirk Island in the past 1–2 million years.

*Robinsonia* is the second largest genus in the archipelago. The largest is *Dendroseris*, also of Asteraceae but from still another tribe (Cichorieae). This latter genus is of interest as it has derived cladogenetically on the older island with three independent dispersals to the younger island and three anagenetic speciations there (Sanders *et al*. 1987; Pacheco *et al*. 1991; Sang *et al*. 1994). Most of these species are quite rare now, however, which precluded our being able to obtain sufficient population data for genetic evaluation. *Robinsonia* has eight species, but two are presumed extinct (*R. berteroi* and *R. megacephala*; Danton *et al*. 2006). Our studies have focussed on five species having originated cladogenetically on the older island. Comments have already been made regarding the one anagenetically derived species (*R. masafuerae*) on Alejandro Selkirk Island. The results from AFLP data are shown in Fig. 3 and from SSRs in Fig. 4. Most notable from the SplitsTree graph in Fig. 3 is that the different species of *Robinsonia* are very distinct genetically. Divergence has obviously taken place during adaptive radiation and also during a maximum time available of 4 million years. The species *R. gayana*, *R. thurifera* and *R. saxatilis* form an evolutionary complex, which taxonomically has been regarded as sect. *Robinsonia* (Skottsberg 1922, as sect. *Symphyolepis*; Takayama *et al*. 2015). *Robinsonia gracilis* ties with *R. evenia* and its close anagenetic relative *R. masafuerae* in sect. *Eleutherolepis* (Skottsberg 1922). With SSR data (Fig. 4), the species are also very distinct and genetically more cohesive, with the anagenetic species *R. masafuerae* showing the greatest genetic diversity. Another important point seen clearly in Figs 3 and 4 is that the range of genetic diversity within each of these cladogenetic species is limited in comparison with the anagenetically derived species discussed above.

Although *Erigeron* is not an endemic genus in the archipelago, six endemic species occur there having evolved via cladogenesis and adaptive radiation. The origin of this complex is unusual in that the colonist(s) presumably arrived directly to the younger island (Valdebenito *et al.* 1992). Amplified fragment length polymorphism and SSR data (Figs 3 and 4) reveal considerable genetic diversity within these endemic species, and each species is reasonably distinct. An exception is the *Erigeron ingae* complex consisting of *E. ingae*, *E. luteoviridis* and *E. turricola*. These species are sometimes difficult to distinguish morphologically. Solbrig (1962) and Marticorena *et al.* (1998), for example, placed *E. turricola* into synonymy with *E. ingae*, but Danton *et al*. (2006) kept them distinct. The molecular data parallel this morphological inconsistency. This may be a population complex in early stages of speciation, now undergoing divergence from within a pool of morphological and genetic variation. All of these species grow in the ‘alpine zone’ on the younger island (Skottsberg 1922), and we have not noticed any clear habitat differences among them. The species *E. rupicola* is confined to coastal rocks along the sea and also penetrates into the quebradas (ravines); its close relative, *E. stuessyi*, is also found on rocky ledges but residing inside the cool and deep ravines. *Erigeron fernandezianus* occurs in a broad altitudinal range (100–1200 m), and it inhabits mainly rocky areas in middle elevation plains, quebradas and ridges. This species also occurs on the older island, but it is found there in many plant communities and especially in disturbed sites. It appears, therefore, to be an example of back migration from the younger to the older island (Valdebenito *et al*. 1992; López-Sepúlveda *et al.* 2015).

Although most species of *Erigeron* on the younger island are distinct genetically, the degree of distinctness is much less than observed among species of *Robinsonia* on the older island (Figs 3 and 4). It may be that these species of *Erigeron* have had less time to diverge in comparison with those of *Robinsonia*. With the passage of time, therefore, the genetic profiles of species undergoing adaptive radiation may remain narrow due to strong directional selection in each different habitat. In both *Erigeron* and *Robinsonia*, however, the range of genetic variation seen is less than that in the anagenetically derived species.

## Discussion

### Comparison of anagenesis and cladogenesis

Predictions from theory (Stuessy 2007) would suggest that higher levels of genetic diversity should be found within the anagenetically derived species. This is because the founding population increases in size over time, accumulating genetic diversity mainly through mutation and recombination. One would expect no (or very little) geographic partitioning over the landscape. Likewise, due to a lack of strong selection, one would not expect to find high levels of private alleles or bands, nor a high RI. With cladogenetic speciation, on the other hand, one would expect less overall genetic diversity within each species, but with more private alleles due to strong directional selection. As for impact from the age of the islands, one would predict less total genetic diversity within anagenetically derived species on the younger island because diversity increases through time. As for the cladogenetic species, one would predict less genetic divergence (distinctness) on the younger island in comparison with species on the older island, because directional selection continues over time and refines the genetic profile of each species as it adapts to the particular ecological zone.

Results from genetic analyses of 5 anagenetic species and 10 cladogenetic species allow comparisons between the two modes of speciation and the two islands of differing ages (Tables 1–3). A number of general points can be observed (Table 4 and Fig. 5). First, in anagenetic species, the level of genetic diversity tends to be higher per species than in the cladogenetic species, especially on Robinson Crusoe Island. This can be seen in percentage of polymorphic bands, SDI, AGDOL, observed proportion of heterozygotes, expected proportion of heterozygotes, number of alleles per locus and allelic richness. Second, in the anagenetic species, the individuals on each island behave genetically as one large population, showing no genetic pattern over the landscape (López-Sepúlveda *et al.* 2013*b*, 2014; Takayama *et al.* 2015; P. López-Sepúlveda, K. Takayama, D. J. Crawford, J. Greimler, P. Peñailillo, M. Baeza, E. Ruiz, G. Kohl, K. Tremetsberger, A. Gatica, L. Letelier, P. Novoa, J. Novak, T. F. Stuessy, submitted for publication). This is true on both islands of differing ages. This suggests that this pattern can develop easily within 1–2 million years and that it can persist for up to 4 million. This is consistent with the results reported for Ullung Island, Korea, which is known to be 1.8 million years old (Pfosser *et al.* 2005; Takayama *et al*. 2012, 2013*a*). Third, the ability of an immigrant population to radiate adaptively has much to do with the properties of the colonists (and progenitors) and less with differences of habitat. Some colonists remain as a single larger population and are not responsive to adaptive change in different ecological zones, whereas others disperse well to micro-zones and quickly become modified morphologically and genetically. Fourth, perhaps most importantly, the total amount of genetic diversity within an anagenetically derived species in comparison with an entire adaptively radiating lineage is approximately the same (Fig. 5).

### Genetics of speciation in endemic plants of oceanic islands

A number of previous studies have assessed levels of genetic variation within and among populations of endemic species of the Juan Fernández Archipelago with other markers such as isozymes, random amplified polymorphic DNA (RAPDs) and inter simple sequence repeats (ISSRs). Isozymes have been analysed in *Dendroseris* (Crawford *et al*. 1987), *Chenopodium sanctae-clarae* (Crawford *et al*. 1988), *Wahlenbergia* (Crawford *et al.* 1990), *Robinsonia* (Crawford *et al.* 1992), *Lactoris* (Crawford *et al.* 1994) and *Myrceugenia* (Jensen *et al*. 2002). RAPDs have been investigated in *Dendroseris* (Esselman *et al*. 2000) and *Lactoris* (Brauner *et al*. 1992), and ISSRs also in *Lactoris* (Crawford *et al*. 2001*b*).

Crawford *et al*. (2001*a*) summarized the results from isozyme studies on 29 endemic species of the Juan Fernández Archipelago, and this represents the best set of observations to compare with the AFLP and SSR data summarized here. The most conspicuous result is that the mean genetic diversities at the species level are low (*H*_es_ = 0.065). Higher levels of diversity were seen in larger populations or in many small populations and also in outcrossing species in contrast to selfers. Of relevance for comparisons to the present study, isozymes have been analysed from four species of *Robinsonia* and in *M. fernandeziana*, *E. fernandezianus* and *R. venustus*. It is difficult to compare the results of the isozymes because they provide less detailed genetic information than from AFLPs and SSRs. Isozyme studies on the endemic *Lactoris fernandezianus*, for example Crawford *et al.* (1994), revealed virtually no variation, but ISSRs showed considerable variation within and among populations (Crawford *et al*. 2001*b*). Studies on isozymes (Crawford *et al*. 1987) and RAPDs (Esselman *et al*. 2000) from *Dendroseris* showed greater resolution of relationships from the latter. The isozyme data for the four cladogenetically derived species of *Robinsonia* show higher levels of genetic variation than in the anagenetic *R. venustus* (Crawford *et al*. 1993*b*) and *Myrceugenia* (Jensen *et al*. 2002), which would be in contrast to the trends documented here. It is important, therefore, that for questions involving population genetics in endemic plants of oceanic islands, rapidly evolving markers need to be used.

The employment of AFLPs and SSRs in the present study from 15 species of the Juan Fernández Archipelago, therefore, does provide detailed genetic data at the population level for purposes of comparing consequences of different modes of speciation. A general review has recently been published on the general topic of interpretation of genetic variation within endemic species of oceanic islands (Stuessy *et al*. 2014), and the present data corroborate ideas summarized there. Clearly, the alternative modes of speciation, anagenesis and cladogenesis result in different genetic consequences. Interpretation of the evolutionary significance of levels of genetic diversity, therefore, must be done in context of type of speciation. As can be seen in the results of adaptive radiation in *Erigeron* and *Robinsonia*, on the young and older islands, respectively, the geological age of the island also matters, as this provides the time dimension in which the evolutionary processes unfold.

Another very significant impact on levels of genetic variation in populations of endemic plants of oceanic islands is that from human activity. Because oceanic islands often have agreeable climates and attractive beaches, people have come to live, play and build homes and apartments, all of which have caused pressures on the native vegetation. In the Juan Fernández Archipelago, for example, people have been living continuously on Robinson Crusoe Island for >300 years (Woodward 1969; Wester 1991). It is not impossible that the species of *Robinsonia* on the older island have suffered some genetic loss due to human activity. Although these species occur either on high ridges or in deep forests, far removed from most persons who live at sea level in the village (San Juan Bautista), incursions into the native forest must have taken place and some plants destroyed. It is known that two species of *Robinsonia*, both on Robinson Crusoe Island, are now extinct (*R. berteroi* and *R. megacephala*; Danton and Perrier 2005; Danton *et al*. 2006). Assessing the level of human impact on the vegetation of an oceanic island, therefore, is challenging. At least in the Juan Fernández Archipelago, there were no aboriginal peoples, and human activity could only have begun with discovery by Europeans (Juan Fernández; Medina 1974) at the end of the 16th century. Since that time, however, considerable negative impact from human activity has been documented in the archipelago (Wester 1991; Matthei *et al.* 1993; Stuessy *et al.* 1997; Swenson *et al*. 1997; Cuevas and Leersum 2001; Greimler *et al.* 2002; Dirnböck *et al*. 2003; Cuevas *et al*. 2004; Ricci 2006; Vargas *et al*. 2011), especially from introduced animals, such as rats, rabbits and goats (e.g. Camus *et al.* 2008). These combined activities have surely had some impact on the levels of genetic variation within and among populations.

## Sources of Funding

This work was supported by an FWF (Austrian Science Fund) grant (P21723-B16) to T.F.S. and a Japan Society for the Promotion of Science (JSPS) Postdoctoral Fellowship for Research Abroad (grant 526) to Ko.T.

## Contributions by the Authors

Ko.T. conceived the idea behind the article; all authors participated in the field work except G.K. and Ka.T.; J.N., P.L.-S., G.K. and Ko.T. completed the laboratory work; J.N. coordinated the NGS data acquisition; T.F.S. and Ko.T wrote the initial draft and all authors contributed to subsequent drafts and offered comments for improvement.

## Conflict of Interest Statement

None declared.
